# Modification of Epoxy Compositions by the Application of Various Fillers of Natural Origin

**DOI:** 10.3390/ma16083149

**Published:** 2023-04-17

**Authors:** Anna Sienkiewicz, Piotr Czub

**Affiliations:** Department of Chemistry and Technology of Polymers, Cracow University of Technology, Warszawska Str. 24, 31-155 Cracow, Poland

**Keywords:** epoxy resin, plastic composite, natural composite fillers, oak wood flour waste, peanut shells

## Abstract

A series of composites based on epoxy resin filled with additives of natural origin were prepared to investigate the influence of such fillers on the properties of the epoxy compositions. For this purpose, the composites containing 5 and 10 wt.% of additive of natural origin were obtained using the dispersion of oak wood waste and peanut shells in bisphenol A epoxy resin cured with isophorone-diamine. The oak waste filler had been obtained during the assembly of the raw wooden floor. The performed studies include testing of samples prepared using unmodified and chemically modified additives. Chemical modification via mercerization and silanization was performed to increase the poor compatibility between the highly hydrophilic fillers of natural origin and the hydrophobic polymer matrix. Additionally, the introduction of NH_2_ groups to the structure of modified filler via 3-aminopropyltriethoxysilane, potentially takes a part in co-crosslinking with the epoxy resin. Fourier Transformed Infrared Spectroscopy (FT–IR), as well as Scanning Electron Microscopy (SEM), were carried out, to study the influence of performed chemical modification on the chemical structure and morphology of wood and peanut shell flour. SEM analyses showed significant changes in the morphology of compositions with chemically modified fillers, indicating improved adhesion of the resin to lignocellulosic waste particles. Moreover, a series of mechanical (hardness, tensile strength, flexural strength, compressive strength, and impact strength) tests were carried out, to assess the influence of the application of fillers of natural origin on the properties of epoxy compositions. All composites with lignocellulosic filler were characterized by higher compressive strength (64.2 MPa—5%U-OF, 66.4%—SilOF, 63.2—5%U-PSF, and 63.8—5%SilPSF, respectively), compared to the values recorded for the reference epoxy composition without lignocellulosic filler (59.0 MPa—REF). The highest compressive strength, among all tested samples, was recorded for the composite filled with 10 wt.% of unmodified oak flour (69.1 MPa—10%U-OF). Additionally, higher values of flexural and impact strength, concerning pure BPA-based epoxy resin, were recorded for the composites with oak filler (respectively, flexural strength: 73.8 MPa—5%U-OF and 71.5 MPa—REF; impact strength: 15.82 kJ/m^2^—5%U-OF, 9.15 kJ/m^2^—REF). Epoxy composites with such mechanical properties might be considered as broadly understood construction materials. Moreover, samples containing wood flour as a filler exhibit better mechanical properties compared to those with peanut shell flour (tensile strength for samples containing post-mercerization filler: 48.04 MPa and 40.54 MPa; while post-silanization 53.53 MPa and 42.74 MPa for compositions containing 5 wt.% of wood and peanut shell flour, respectively). At the same time, it was found that increasing the weight share of flour of natural origin in both cases resulted in the deterioration of mechanical properties.

## 1. Introduction

Intensive development of science and various technological methods, together with intensively growing demand for modern construction materials, as well as increasing awareness, and the resulting legal changes regarding the use of materials from renewable energy sources, are currently the main driving factor of progress in modern science. Epoxy resins appeared on the market in the thirties of the last century. Since then, continuous work has been underway to improve the methods of the synthesis and functional properties of epoxy materials. There is constant research on, e.g., innovative production solutions [1,2], greener synthesis routes [3,4,5], and new ways of cross-linking [6,7,8]. The studies of pro-ecological methods of epoxy resin synthesis, among others, concern, the use of raw materials and synthesis methods towards reducing or complete elimination of bisphenols from raw materials and amines—from hardeners [9,10]. In general, epoxy resins are characterized by valuable properties such as good mechanical strength, thermal stability, and polarity, thanks to which epoxy resins exhibit good adhesion to other materials, including glass, concrete, ceramics, and metals. Epoxy resins, due to their mentioned advantages are used as semi-finished products, such as adhesives, varnishes, binders, putties, and sealants, as well as independent products, i.e., powder paints, saturators in electronics, binding masses, and enamels [11]. In addition, epoxy resins, mainly cast, are used to produce laminates, which are widely applied, from electronics to construction materials for the automotive, aviation, and shipbuilding industries [12]. However, despite the mentioned competitive properties, cured epoxy compositions are stiff and brittle, which makes them difficult to use as construction materials. There are many ways to improve these physical properties. One of them is the addition of a filler to the epoxy matrix to obtain composites. Fillers are used in epoxy resin systems mainly for reducing production costs and improving the mechanical properties of these materials. In addition, a wide range of fillers used for epoxy resins plays an important role in modifying thermal properties. Moreover replacing part of the resin with a certain amount of the filler allows for the limitation of the exothermic effect accompanying the curing reaction of the epoxy resin and increases the thermal conductivity of the final composition [13]. It is worth highlighting here that companies are continuously seeking new materials and improved processes to produce better products to maintain competitive advantage and increase profit margins. Fibers of natural origin are cheaper, lighter, and better in terms of environmental protection as an alternative to synthetic fibers in polymer composites. This environmental aspect is in line with “green” chemistry, thus increasing the attractiveness of natural fiber in the market.

Currently, on the market epoxy resins are largely intended for use as composites. It is worth noting that, there are several different fillers available for such plastics, both of natural origin, such as sisal fibers [14], jute flour [15], as well as mineral fillings limestone, [16,17], chalk [18], and quartz dust [19]), and synthetic (e.g., PET [20,21], aramid [22], and graphite fibers [23]). Each of the already tested fillers within the epoxy composite affects different materials’ properties resulting in various possible applications. In the case of using fillers for epoxy resins, currently the most common are glass or carbon fibers (mats and short-cut fibers). However, the growing pro-environmental awareness of society also influences the change and intensity of work related to the research and application of natural raw materials in technological processes. A good example of such a tendency is the automotive industry, whose content of ecological materials of natural origin or recyclates is legally regulated. These regulations state that vehicles should contain up to 95% of reusable materials. In addition, in recent years, more popular are also social movements aimed at conscious consumerism, e.g., among others, to pay attention to the content of ecological materials in purchased products. These trends are followed by work on adapting plant fibers to a wider application and improvement of their functional properties. The world production of natural fibers was estimated at 33.7 million tonnes in 2022, which was over one million greater than in 2021 [24]. The most commonly used natural fillers for polymeric materials are cellulose materials, lignin and lignocellulosic (e.g., wood flour), natural fibers (e.g., sisal or jute), and modified starch. The mechanical properties of natural fibers vary greatly from industrially used synthetic fibers, but due to their smaller size density, price, and biological origin, they are very attractive in terms of use in new technological solutions. The application of natural origin waste materials, i.e., walnut shells, hazelnut shells, and sunflower or wood flour has received a lot of attention recently. In the presented studies we attempted to apply and test the effect of the introduction of natural fillers, in the form of groundnut flour and oak flour from the processing of wooden floors into bisphenol A epoxy resin.

Peanut (*Arachis hypogaea* L.) is an oil legume grown throughout the world, mainly in tropical climates. Global peanut production is approximately 47 million tonnes, of which China is the largest producer with about 17 million tonnes of production per year [25]. Peanuts are a popular food product, both in the form of pure fruit, as well as after processing. Unfortunately, the processing process produces many waste products in the form of shells, skins, and flour. There are about 230–300 g of shells per kilogram of peanuts, while the skins make up less than 3 wt.% of peanuts. During the production of oil from peanuts, a by-product in the form of flour is produced. The waste product is mainly intended for animal fodder [26]. Peanut shells are considered an agro-industrial waste with a slow degradation rate under natural conditions [27]. They are composed of 35.7% cellulose, 18.7% hemicellulose, and 30.2% of lignin, as well as a small number of proteins, sugars, fats, and minerals. In the face of such a substantial amount of bio-waste from peanuts, there are numerous studies carried out to develop new ways of waste management.

Adhikari et al. [28] conducted studies on antioxidant activity and amino acid content in the shells of Korean peanut varieties. The content of polyphenols was 428.1–739.8 mg of gallic acid equivalent per g (mg equivalent quercetin/g), flavonoid content 142.6–568.0 mg equivalent quercetin/g, and amino acids 5.76–34.56 mg/g. It has been proven that due to the significant content of polyphenolic compounds, as well as the presence of flavonoids, the shells have antibacterial properties and can also be a natural source of antioxidants in the food and cosmetics industry.

An important use of waste shells is the production of bio-products, such as liquid biofuels [29,30]. Biodiesel was synthesized, e.g., by the fungi *Aspergillus niger* from peanut shells during a process catalyzed by lipase [31]. The resulting product was characterized by greater viscosity compared to biodiesel made from waste sunflower oil, but on the other hand, it was more resistant to low temperatures. It is worth noting that in the production of bioethanol from peanut shells, a pre-treatment in the form of saccharification of biomass is necessary, aimed at converting cellulose into simple sugars, which are needed for alcohol fermentation. Bioethanol obtained by the fermentation of yeast *Saccharomyces cerevisiae* can be used in gasoline engines.

Nutshells can also be used to produce paper [32,33]. For this purpose, bio-waste is crushed and subjected to alkali and sulfate pulping, then washing, bleaching, drying, and compressing into sheets of paper.

Another way to manage peanut bio-waste is to use it as a biosorbent for the adsorption of heavy metals from wastewater. Shruthi and Pavithra [34] stated that during biosorption using peanut waste, which might effectively be used in small-scale treatment plants, the pH is the most important factor affecting the biosorption potential. The maximum adsorption was achieved in an acidic environment. They achieved the removal efficiency for Cu (II) and Pb (II) using groundnut shell powder at a level of 68.2% and 77.8%, respectively, for the optimum dosage of 50 g for copper and 30 g for lead. Biosorption is a cheaper and more effective method than conventional technologies including membrane, precipitation, or ion exchange. Performed studies indicate that it is possible to obtain maximum adsorption of copper ions at the level of 93.9% at pH 6, lead ions up to 77.8%, and there is also observed effectiveness in removing zinc and chromium ions [35,36].

Additionally, due to their chemical structure, research is also conducted in the direction of the use of shells as additives to improve the strength properties of polymeric materials. Samson et al. [37] synthesized polylactide nanofibers reinforced by groundnut shell particles of grain size 150 µm. In endurance tests, the properties of PLA fibers reinforced with shells showed that in the case of application of unmodified peanut shells, the best properties were observed for material containing 5–6 wt.% of filler. For such composite material, stiffness of 25 MPa, stress at the breaking of 0.21 MPa, and impact strength of 0.057 J was registered and compared to the stiffness of 1 MPa, stress at the break—0.01 MPa, and impact strength—0.055 J noted for non-reinforced polylactide fibers. Such PLA fibers, reinforced by additives of natural origin, may have potential applications in tissue engineering and drug delivery systems. Obasi [38] used peanut shells as fillers for the polymer matrix made of low-density polyethylene. The bio-waste had been ground into fine-sized particles of 300 µm, additionally, a coupling agent in the form of polyethylene maleate was used to modify the surface of the filler. The filler was melt mixed with the matrix in the extruder at 120–150 °C. As indicated, the addition of a coupling agent increased the adhesion between the filler and the matrix, which resulted in reduced water absorption and swelling of the plastic, as well as less composite weight loss due to enzymatic degradation in comparison with a composition devoid of polyethylene maleate. In addition, together with increasing the percentage of filler in the polymer composite improved mechanical properties (flexural strength, Young’s modulus, impact strength, and hardness) were noted. To an even greater extent, as in the above-cited example of using waste peanut shells as fillers for polymeric materials, waste wood flour is applied. It is characterized by low weight and density, susceptibility to biodegradation and recycling, as well as good strength properties [39]. The addition of wood flour can affect not only the strength of composite materials, but also contributes to the change of glass transition temperature, deformation of final products, or improve their thermal stability [40,41].

As indicated above, the research presented within this paper focused on the comparison of the effect of the application of different fillers of natural origin on the properties of epoxy resin composites. In the role of fillers, we used the waste oak flour from the processing of parquet flooring and waste peanut shells flour. The Authors obtained composites containing 5 and 10 wt.% of additive of natural origin using the dispersion of oak wood waste and peanut shells in bisphenol A epoxy resin cured with isophorone-diamine. The presented research discusses the influence of two-stage modification via the mercerization and silanization process on the chemical structure and morphology of the filler particles. Performed chemical modification of additives if natural origin were performed to increase the poor compatibility between the highly hydrophilic fillers of natural origin and the hydrophobic polymer matrix. Fillers and the obtained epoxy composites were analyzed using Fourier Transformed Infrared Spectroscopy (FT–IR), as well as Scanning Electron Microscopy (SEM). Moreover, a series of mechanical tests including hardness, tensile strength, flexural strength, compressive strength, and impact strength were carried out, to assess the influence of the application of fillers of natural origin on the properties of epoxy compositions. To the best of the Author’s knowledge presented research for the first time undertakes on the possibility of the application of oak waste filler, which has been obtained during the assembly of raw wooden floor planks for the parquet flooring, in bio-materials based on bisphenol A epoxy resin. In light of mentioned above the scale of the production of such waste, it is very important to investigate the different possible ways of natural waste management.

## 2. Materials and Methods

Materials. Epidian 6 (low-molecular-weight bisphenol A epoxy resin), EP6, CIECH Sarzyna S.A., EV = 0.536 mol/100 g; IDA hardener (amine hardener based on isophorone-diamine for liquid epoxy resins, CIECH Sarzyna S.A.), the oak wood waste flour (FHU Parkiety Smolik), and peanut shells (MAKAR Bakalie).

### 2.1. Natural Filler Preparation

#### 2.1.1. Initial Preparation

(1) The oak wood flour, obtained as post-production waste, during the sanding of wooden floors, was separated into homogeneous fractions using a laboratory shaker for sieve analysis. The obtained fractions (>0.2 mm; 0.2–0.16 mm; 0.16–0.1 mm; 0.1–0.076 mm; 0.076–0.056 mm; 0.056–0.04 mm, and <0.04 mm) were used for the analysis. The oak wood flour was pre-dried at a temperature of 60 °C for 48 h and subjected to chemical modification.

(2) Peanut shells—Peanuts, as waste after processing raw nuts for the food industry, were cracked, and then the shells were separated from the husks. The shells were pre-ground in an electric mill to obtain particle sizes <0.04 mm. The obtained peanut shell flour was pre-dried at a temperature of 60 °C for 48 h and subjected to chemical modification.

#### 2.1.2. Chemical Modification of Waste Filler

**Figure 1:**
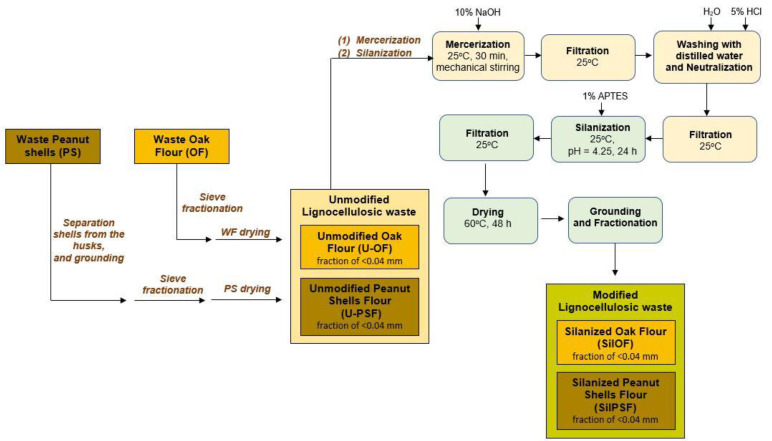
Block diagram of the chemical modification process of lignocellulosic waste.

In the first stage of the performed research, the natural waste, in the form of oak wood flour, and peanut shells, was prepared for further application (Figure 1).

The wood filler, obtained as post-production waste, during the sanding of wooden floors, was separated into homogeneous fractions using a laboratory shaker for sieve analysis. Peanuts were cracked, and then the shells were separated from the husks and preliminarily ground in an electric grinder. Then the natural fillers were pre-dried at a temperature of 80 °C for 48 h and next subjected to the two-stage chemical modification on the way: (1) mercerization using 10% NaOH solution, followed by (2) silanization with 3-aminopropyltriethoxysilane (APTES). The procedure for the modification process for both fillers was analogous.

**Figure 2:**
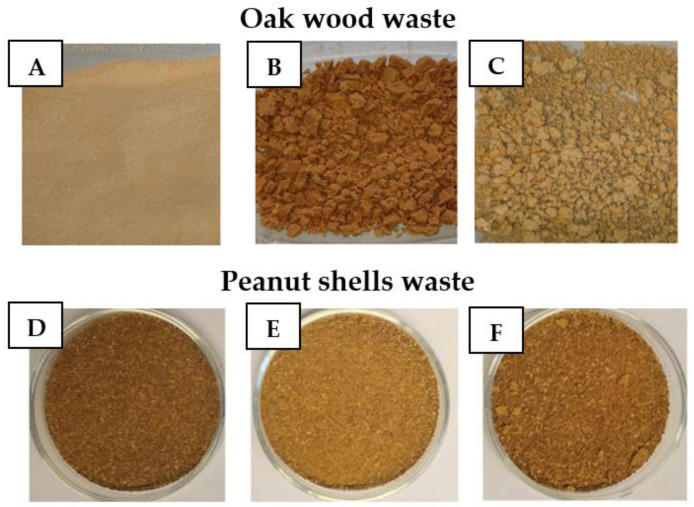
Lignocellulosic filler for the application in epoxy composites: (**A**) unmodified waste oak flour; (**B**) mercerized oak flour; (**C**) silanized oak flour; (**D**) unmodified peanut shells flour; (**E**) mercerized peanut shells; and (**F**) silanized peanut shells flour.

(1) Mercerization—The natural filler in an amount of 150 g was placed in a beaker and hot distilled water was added to rinse the filler of the industrial contaminations. Then, during the mercerization process, the lignocellulosic additive was immersed in a 1000 mL of 10% solution of NaOH for 30 min, the obtained suspension was vigorously stirred for 30 min using a mechanical stirrer, left to decant and then the modified precipitate was filtered on a Büchner funnel. It was observed that in both cases the solutions in which fillers were immersed changed color from transparent to dark brown. Moreover, the change in the color of lignocellulosic flour was observed, which could indicate the loss of hydroxyl groups and the reduced content of cellulose-derived carbonyl bonds (Figure 2). Moreover, such a phenomenon was also noticed during the process of the degradation of lignin, caused by the neutralization of wood flour [42]. Additionally, significant swelling of filler was observed. It was especially noticed in the case of peanut shells, which, after mixing a thick layer, formed by lighter particles of ground shells were of the liquid. It made the decantation much more difficult and time-consuming. Moreover, during the filtration process on a Büchner funnel, it was necessary to replace the filter paper several times, due to the clogging of the pores of the used filter by mercerized lignocellulosic waste. Then the waste was washed with distilled water and neutralized using a 5% hydrochloric acid solution. The mercerized waste was dried at 80 °C for 48 h. The dried waste was then ground and re-fractionated using a laboratory shaker for sieve analysis.

(2) Silanization—In a 1000 mL volumetric flask, a 1% solution of 3-aminopropyltriethoxysilane (APTES), in a mixture of distilled water and methanol in a 1:1 volume ratio, was prepared. Then the wet, mercerized filler was transferred to the beaker and subjected to a silanization process using 500 mL of prepared 1% solution of 3-aminopropyltriethoxysilane in a mixture of distilled water and methanol. The resulting solution of APTES was acidified by adding 290 mL (in portions of 10 mL) of 5% acetic acid to pH = 4.23. It was found that in a suitably acidic environment, the silane under the influence of water is hydrolyzed. During this reaction, alkoxysilanes and alcohols are produced. The silane groups condense to form a siloxane. This is due to the occurrence of hydrogen bonds between the silane groups and leads to the formation of the so-called siloxane bridges. During the contact of siloxanes with the surface of natural fibers, they are substituted for hydrogen atoms by hydroxyl groups [43].

After adding the silane solution to the filler, the mixture was stirred intensively using a mechanical stirrer for 30 min and left for 24 h, and then the waste was washed with distilled water and filtered on a Büchner funnel. Moreover, in the case of silanization, a change in the color of the liquid above the precipitate from transparent to caramel was observed. Moreover, shells and wood flour subjected to this modification swelled, but significantly to a lesser extent than that observed during the mercerization process. Moreover, just like before, during the silanization, a layer of lighter ground peanut shells appeared on the surface of the liquid, but there were fewer of them than in the case of mercerization. Additionally, it was observed that filtration of the modified sludge was much faster than in the case of filler modified in the first stage.

The modified lignocellulosic waste products in the form of silanized oak flour (SilOF) and silanized peanut shells flour (SilPSF) were dried at a temperature of 60 °C for 48 h. The time of drying was established based on the determination of change in the weight of the waste due to moisture evaporation. When this process of evaporation was complete, the process of drying was finished and the wood filler was ground and subjected to fractionation to finally obtain lignocellulosic filler with a size <0.04 mm.

### 2.2. Epoxy Composites with Modified Wood Waste

The weighed amount of low molecular weight epoxy resin Epidian 6 containing 0.536 mol/100 g epoxy groups and wood waste (in the amount of 5 and 10 wt.%) was thoroughly mixed using a mechanical mixer (700 rpm) for 30 min. Then the proper amount of hardener (isophorone-diamine, IDA) and deaerating agent (BYK A530, 1 wt.%. concerning the total weight of the composition) was added, followed by mixing the entire composition for an additional 5 min using a mechanical mixer (1000 rpm). Next, the composition was de-aerated for 3 min at a pressure of 0.8 MPa. Finally, the composition was poured into Teflon molds. Since the planned performance of mechanical tests was following applicable standards (described below within Section 2.5) the curing process of the obtained composites was carried out in molds in the form of paddles, beams, and rollers at room temperature for 24 h. After that time the obtained samples were taken out of the molds and subjected to seasoning in the lab temperature for 7 days and finally, they were subjected to additional crosslinking at the temperature of 80 °C (24 h).

### 2.3. Spectroscopic Measurements

The changes in chemical structure, which have arisen under the influence of the performed mercerization and silanization of wood waste were recorded using a Nicolet iS5 spectrometer equipped with a diamond crystal in an attenuated total reflectance unit manufactured by Thermo Electron Corporation (Madison, USA with Labsoft, Warsaw as representation in Poland). All analyses were carried out at room temperature. First, the small amount of pre-dried filler in a form of fine-grained waste flour was transferred onto the crystal attenuated total reflectance unit (iD7 ATR, Thermo Fisher Scientific, Waltham, USA with Labsoft, Warsaw as representation in Poland). Followed by pressing the filler with a spatula to cover the crystal with an even layer of the analyzed sample. Then, the waste flour was additionally pressed down with a screw equipped with an attachment for loose samples to tightly compress the sample of filler. Spectra were recorded for wavenumbers in the range of 4000–600 cm^−1^ in 4 cm^−1^ intervals. Sixteen scans were averaged. Within the paper, registered spectra are presented using the dependence of transmittance T (%) and wavenumber v (cm^−1^). The obtained results were processed using the Omnic 9.6 computer program.

### 2.4. Morphological Analysis

A morphological analysis of the natural fillers and the obtained epoxy composites was conducted using a JEOLJSM-6010LA scanning electron microscope (Tokyo, Japan) at a 5 kV acceleration. The analysis of post-chemical modification morphological changes within the natural filler was performed on pre-dried flour particles. While, in the case of the samples of epoxy composites, SEM micrographs were recorded of the impact-fractured surfaces of the cured compositions. The approximate dimensions of such samples were 2 mm × 10 mm. Regardless of the form of the analyzed sample, each of them was coated with a thin film of gold. Microphotographs were recorded in jpg format and an InfranView 4.57 graphic program was used for their further processing.

### 2.5. The Mechanical Properties

The prepared compositions were tested on Zwick 1445 apparatus (Wroclaw, Poland) using samples in the form of paddles, beams, and rollers, which were prepared according to the standards: PN–EN ISO 527-1:2012 (the tensile strength, elongation at break, and modulus elasticity); PN–EN ISO 178:2011 (the flexural strength, elasticity, flexural modulus); PN–EN ISO 604:2006 (the compressive strength and compression set); PN–EN ISO 868:2005 (hardness in Shore A, the InSize apparatus); PN–EN ISO 179-2:2001 (the impact strength without notches by the Charpy method; and ZORN PSW 4J Digital apparatus.

The prepared epoxy compositions were poured into Teflon molds in the shape of paddles, beams, and rollers, which after curing over 24 h in molds, were seasoned in the lab temperature for 7 days, then crosslinked for 24 h at the temperature of 80 °C. In the final step of the preparation, the surface of the samples for mechanical tests was leveled using a hand grinder.

To measure the tensile strength, elongation at break, and modulus of elasticity, samples in the form of paddles (type B) with the measurement section of dimensions: 4 mm× 10 mm (at cross section) and 50 mm (length), were used. Such a sample was placed between tensile jaws and tests were conducted at 5 mm/min testing speed. Flexural strength, elasticity, flexural modulus, and deflection were measured using samples in the form of cuboid beams with cross-section dimensions of 4 mm× 10 mm, additionally applying 64 mm spacing between supports and a testing speed of 10 mm/min. The compressive strength and compression set were tested using samples in the form of rollers with 10 mm diameter and 25 mm height. The rollers were placed between two compressive jaws in the form of metal plates. All the above tests, using Zwick 1445 apparatus, were performed at least five times, and the obtained results were averaged. The compression was performed by applying the testing speed of 0.8 mm/min.

Hardness on the Shore A scale was tested according to PN–EN ISO 868:2005 standard with the use of the InSize apparatus (Bizkaia, Spain). The test is based on the measurement of the resistance posed by a sample when the sharp needle of an indenter is inserted into the sample placed on the glass base of the measuring device. For each tested composite a minimum of ten measurements were taken at different points on the sample at least 6 mm apart and the average value was calculated.

Impact strength without notches was measured by using a Charpy hammer. At the beginning of the test, the sample in the form of cuboid beams with cross-section dimensions of 4 mm× 10 mm was placed at the bottom of the swinging arm which at that point was raised and immobilized. Then, the arm was released and during its free-falling struck the sample at the bottom. Finally, the measured value of energy used for the fracture of the analyzed sample was used for the calculation of the value of impact strength. Tests were performed on a minimum of five samples of each epoxy composite, and the average value was calculated.

## 3. Results and Discussion

### 3.1. Chemical Modification of Waste Filler and the Analysis of Its Chemical Structure

**Figure 3:**
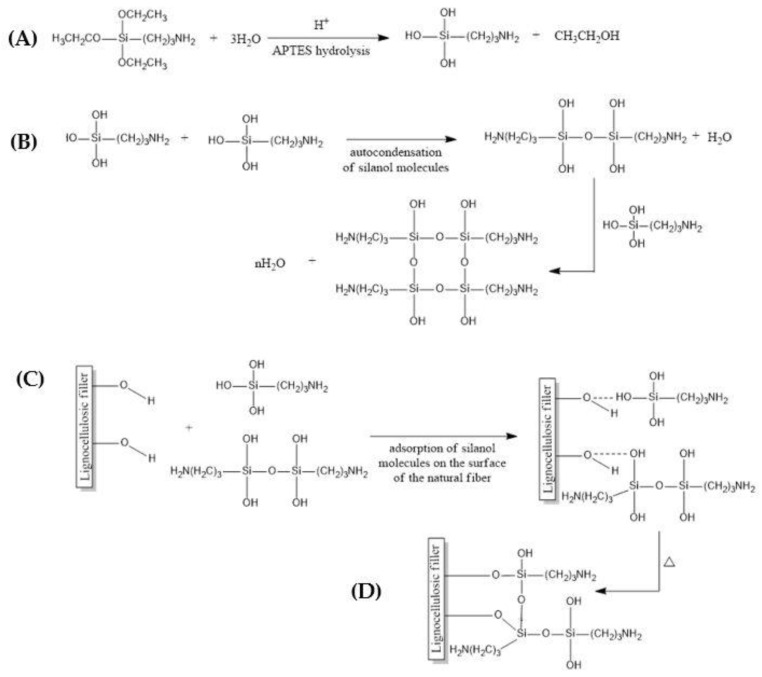
Silanization of lignocellulosic waste using 3-aminopropyltriethoxysilane. (**A**) the hydrolysis of silane molecules; (**B**) self-condensation of silanol molecules; (**C**) the adsorption of silanol molecules on the surface of the lignocellulosic filler; (**D**) the grafting of silanol molecules.

The chemical modification of waste fillers for further application as reinforcement to bisphenol A epoxy resin was performed via the silanization process proceeded by washing with distilled water and the mercerization of lignocellulosic waste. Generally, in the first stage of silanization, silane hydrolysis occurs, resulting in the formation of silanol and alcohol. Then silanol reacts with hydroxyl groups of natural fibers forming permanent and stable bonds (chemisorption of silicon compounds on the fiber surface) [43]. The entire process of silanization using 3-aminopropyltriethoxysilane (APTES) might be divided into the following stages (Figure 3):

- The first step is hydrolysis, during which the silane molecules hydrolyze in the presence of water as a catalyst, releasing alcohol and silanol (Figure 3A).
- Self-condensation of silanol molecules is also a possible reaction, however, during the silanization, the process of auto-condensation is considered a side process (Figure 3B).

While conducting the modification, it is important to maintain the conditions of the reaction as competitive, for the silanization itself. Therefore, an acidic environment during most of the reaction time is not conducive to coupling, leading to the acceleration of hydrolysis and inhibition of auto-condensation.

- Next, the adsorption of silanol molecules on the surface of the natural fiber occurs. It is important to mention here that during that time, not only silanol monomers but also silanol oligomers can undergo the adsorption reaction. In addition, silanol molecules adsorbed on the surface can react with subsequent silanol molecules to form polysiloxane structures (Figure 3C).
- Finally, the last step of the process is the grafting of silanol molecules adsorbed on the surface of natural fibers. The grafting process occurs under the influence of the raised temperature, which causes dehydration of the structure.

**Figure 4:**
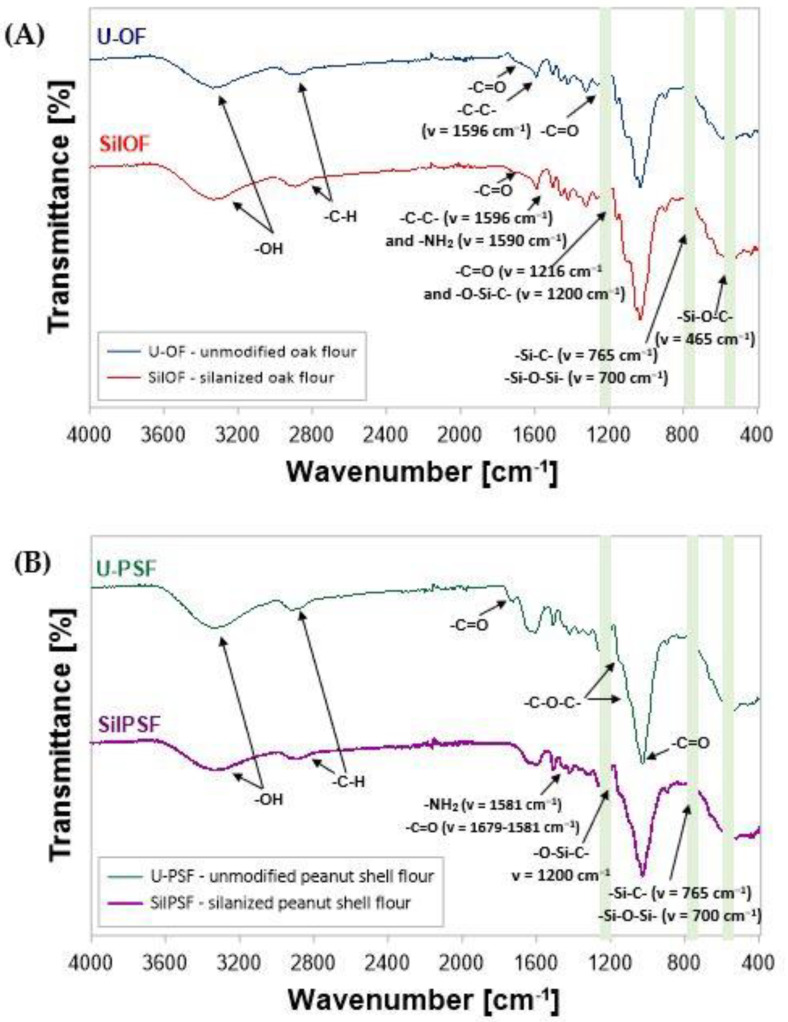
FT–IR spectrum of unmodified and chemically modified fillers in a form of (**A**) waste oak flour (U-OF—unmodified oak flour; SilOF—silanized oak flour) and (**B**) peanut shell flour (U-PSF—unmodified peanut shell flour; SilPSF—silanized peanut shell flour).

The modified products via the silanization process, and also unmodified fillers in a form of oak and peanut shell flour were analyzed both using spectroscopy measurements and capturing SEM microphotographs. The spectrum (Figure 4, Table 1) presents the changes in the chemical structure, which were revealed after the performed modification of the bio-based fillers.

In spectra recorded for unmodified and modified fillers, the characteristic wide bands can be seen corresponding to the stretching vibrations of the -OH group and hydrogen bonds of hydroxyl groups in the range of about 3100–3600 cm^−1^ [44]. On the other hand, in all those analyzed samples of lignocellulosic wastes that have undergone chemical modification, a relatively reduced band intensity was observed in this scope. The changes observed in the discussed area may prove effective in reducing the number of hydroxyl groups contained in lignocellulosic waste, after modification. Moreover, scissors’ deformation vibrations were observed in the spectra of silanized fillers (SilOF and SilPSF) derived from the -NH_2_ moiety. The signal at v = 2878 cm^−1^ is most likely a characteristic band of vibrations stretching -C-H from the -CH and -CH_2_ groups of cellulose and hemicellulose [45]. On the other hand, the signal at v = 1724 cm^−1^ can be attributed to the carbonyl group -C=O of stretching vibrations of the carboxyl group in lignin or the ester group in hemicellulose [46]. At the same time, it should be noted that this signal was not recorded for silanized wood flour. A similar effect was observed by Sgriccia et al. [47], attributing it to the removal of hemicellulose fragments from the surface of the lignocellulosic particles. Signals of relatively low intensity at v = 1592 cm^−1^ and 1470 cm^−1^ are attributed to vibrations of the skeleton -C=C- of the aromatic ring in the lignin structure [48]. On the other hand, the signal at v = 1425 cm^−1^ is related to the symmetrical bending of the -CH_2_ group present in cellulose [47]. The next two signals, at v = 1314 cm^−1^ and v = 1363 cm^−1^, are characteristic of the bending vibrations of the -C-H and C-O groups of the aromatic ring in polysaccharides [49]. The signal at ν = 1254 cm^−1^ in the SilPSF spectrum most likely corresponds to the stretching valence vibrations of the -C-N groups. Moreover, in the SilPSF spectrum, the signal at ν = 1033 cm^−1^ of relatively low intensity can be attributed to valence deformation vibrations of -Si-O. In turn, those at v = 1227 cm^−1^ were most likely recorded as stretching vibrations -C-O of the acetyl group in lignin. In addition, it should be noted that the signal at v = 1227 cm^−1^ is relatively smaller for silanized particles than that recorded for unmodified flour. This signal corresponds to the stretching vibrations of the lignin-derived acetyl group. As a result of the chemical modification, lignin is partially removed from the lignocellulosic waste [50]. Therefore, in the spectrum of silanized fillers, this signal is characterized by a relatively lower intensity, compared to the signal recorded for unmodified flour. The next two signals for wood particles at v = 1106 and v = 1055 cm^−1^, and peanut shell fillers in the range 1021–1018 cm^−1^, are related to the stretching vibrations of the -C-O-C- ring of pyranose present in polysaccharides [51]. In addition, it is also worth mentioning that the signal at v = 1028 cm^−1^ can be attributed to the -C-O stretching vibrations of the hydroxyl groups and ethers in cellulose [45]. In turn, the signal of relatively low intensity at v = 891 cm^−1^ is associated with the presence of β-glycosidic bonds in monosaccharides, while the one at v = 560 cm^−1^ corresponds to the bending vibrations of the -C-OH group [52]. Modification of lignocellulosic particles via silanization by APTES is particularly visible in the FT–IR spectrum at v = 1200 cm^−1^—characteristic vibrations of the Si-O-C group; v = 1050 cm^−1^ and 700 cm^−1^—Si-O-Si vibrations; v = 765 cm^−1^—symmetrical Si-C stretching vibrations and v = 465 cm^−1^—asymmetric bending vibrations Si-O-C. Some of them overlap with the vibrations of the bonds of the complex chemical structure of the lignocellulosic flour [53].

### 3.2. Preparation of Epoxy Compounds Reinforced by the Addition of Oak or Peanut Shell Flour

In the next stage nine compositions with different content of filler in the form of unmodified and modified oak and peanut flour were prepared (Table 2).

During the preparation process, changes in the color and viscosity of individual compositions were observed. The reference mixture was colorless, and the compositions with filler were light brown. At the time of preparing the composition, it was found that the reference mixture containing the Epidian 6 epoxy resin and the amine hardener had the lowest viscosity, while a significant increase in the viscosity of the composition was observed with the increase in the filler content (Please see the Supplementary Materials, Figure S1). In addition, during the pouring of the mixtures, significant changes in the time of cross-linking of individual compositions were found. The visible crosslinking process of composites with a filler share of 10 wt.% started much earlier than in the case of reference samples or composites containing 5 wt.% of filler. Assessment of the degree of cross-linking of the epoxy composition was carried out based on decay analysis bands of epoxy groups at 915 cm^−1^ wavelength, characteristic of group valence vibrations epoxy in the uncured resin (Please see the Supplementary Materials, Figure S2).

### 3.3. Results of Mechanical Tests of Epoxy/Oak (Peanut Shell) Flour Composites

**Figure 5:**
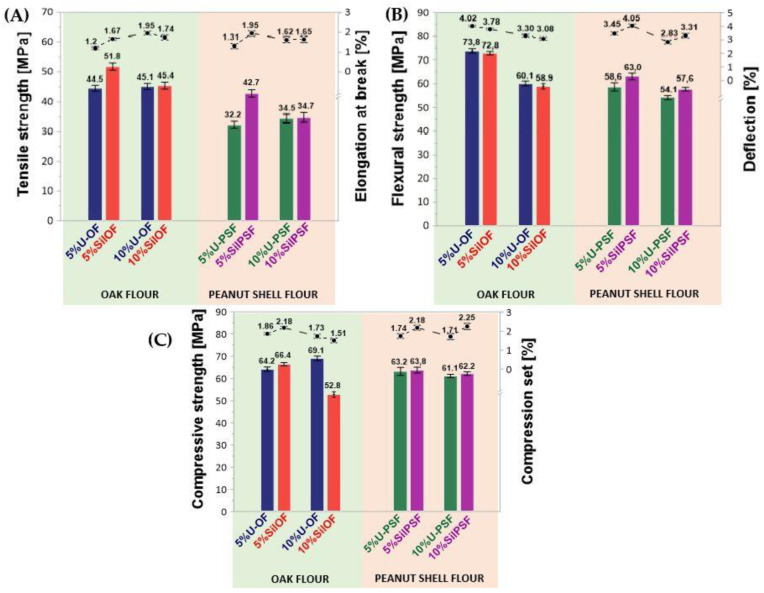
Mechanical properties of compositions based on Epidian 6 filled with wood or peanut shell flour: tensile strength and elongation at break (**A**), flexural strength and deflection of compositions (**B**), and compressive strength and compression set (**C**).

In the next stage, the influence on selected mechanical properties of the introduction of lignocellulosic waste into the epoxy matrix was analyzed (Figure 5, Table 3) using tests, such as the tensile strength, elongation at break, modulus elasticity, flexural strength, elasticity flexural modulus, deflection, compressive strength, compression set, Rockwell hardness, and the impact strength without notches using the Charpy method. Moreover, selected cured composites were subjected to additional analysis including the contact angle, and swelling behavior (Please see the Supplementary Materials, Figures S3–S5).

The reference epoxy composition, without the lignocellulosic filler (REF), was characterized by the best static tensile strength (52.73 MPa) and the highest elongation at break (3.39%). The addition of unmodified lignocellulosic waste, both in a form of oak and peanut shells flour, of size <0.04 mm in the amount of 5% by weight had a significant impact on the deterioration of tensile strength and reduction elongation at break (44.5 MPa, 1.2% for U-OF, and 32.2 MPa, 1.31% for U-PSF, Figure 4A). The recorded lower values of tensile strength values are associated with the hydrophilicity of wood fibers. When comparing the results recorded for oak flour versus peanut shell flour, the worse results for shells may be related to the much higher coverage of the filler particles with pectin and wax compounds. On the other hand, in the case of compositions containing lignocellulosic flour subjected to a two-stage silanization process an improvement in tensile strength and relative elongation was found (respectively, 51.8 MPa and 1.67%—for compositions containing 5 wt.% SilOF and 42.7 MPa and 1.95%—SilPSF). Simultaneously, when comparing composites with different content of modified filler, regardless of the type of used addition SilOF or SilPSF, recorded values for samples containing 5 wt.% were the highest among all tested samples of lignocellulosic epoxy composites. On the other hand, among all samples, the highest values of modulus were recorded for compositions containing 5 wt.% of peanut shell flour, 1618.2 MPa—5%SilPSF, and 1467.4 MPa—5%U-PSF, respectively. At the same time, the value of recorded Young’s modulus for 5%SilPSF was the highest among all samples including the reference composition, for which the recorded value was at the level of 1021.7 MPa. In turn, the smallest value of Young’s modulus was recorded for the 10%SilPSF composition. Based on the obtained results, it can be concluded that the composite 5%SilPSF is characterized by the highest stiffness of its structure. The change in strength properties comparing composites containing silanized and unmodified filler may result in the participation of additional amine groups from the introduced APTES (via the silanization process), which possibly also can take a part in the cross-linking process of epoxy resin. Signals in the form of scissors’ deformation vibrations from introduced amine groups were observed in the FT–IR spectra registered for both silanized fillers (SilOF and SilPSF) (Figure 4). This way supplementary bonds be formed between the modified structure of natural fiber and the polymer matrix. Increasing the cross-linking density caused by the presence of an additional cross-linking agent increased the stiffness of the structure, as indicated by an increase in the value of the modulus. It was also observed that regardless of the applied type of used filler and performed or not chemical modification, with the increase in the filler content in the compositions, a decrease in the value of the tensile modulus was observed.

Based on the test of flexural strength (Figure 4B), the composites containing oak filler were characterized by better mechanical properties. Values of flexural strength, recorded for samples with both unmodified and silanized oak flour were higher than those recorded for reference samples without lignocellulosic filler (71.5 MPa—REF). Among the shell-filled compositions, the highest flexural strength was noted for the composition containing 5 wt.% of waste, subjected to two-stage silanization (63.05 MPa—5%SilPSF). At the same time, for the 5%SilPSF composition, the highest flexural strain value was recorded among all compositions containing peanut filler. It is worth noting here that comparing values of elasticity flexural modulus, most of the tested compositions containing natural filler were characterized by values at a comparable level. Among samples with oak filler, the highest value was recorded for a composition containing 5 wt.% of silanized oak flour (1832.5 MPa—5%SilOF), while for peanut shell composites those with 10 wt.% of unmodified peanut flour (1572.3 MPa—10%U-PSF). Additionally, for the 10%SilPSF composition, the lowest Young’s modulus value of 1159.0 MPa was recorded among all the tested samples.

All composites with lignocellulosic filler were characterized by higher compressive strength compared to the values recorded for the reference epoxy composition without lignocellulosic filler (59.0 MPa—REF). The highest compressive strength, among all tested samples, was recorded for the composite 10%U-OF. While other filled composites were characterized by comparable values of compressive strength (64.2 MPa—5%U-OF, 66.4 MPa—5%SilOF, 63.2 5%U-PSF, 63.8 MPa—5%SilPSF, 61.2 MPa—10%U-PSF, and 62.2 MPa—10%SilPSF, respectively). The highest compressive strain value was recorded for the 10%SilPSF composition (2.25%), while the lowest value was shown for the composition containing 10 wt.% of unmodified peanut shell flour (1.71%—10%U-PSF). Among all tested composites, the compositions filled with 5 wt.% of oak flour were characterized by the highest values of impact strength. Increasing the amount of wood filler resulted in a significant reduction of the impact toughness (15.82 kJ/m^2^—5%U-OF and 15.43 kJ/m^2^—5%SilOF). In the case of composites containing peanut shells, the worst impact strength was recorded for the composition 10%U-PSF.

E. Garcia et al. [54] studied the effect of the addition of 2, 4, 6, 8, and 10 wt.% peanut shells to HDPE. Composites filled with shells were characterized by lower tensile and flexural strength compared to the composition without the filler. At the same time, a slight increase in the hardness of composites with the addition of shells was observed. A difference in the tendency of increasing the value of the modulus of elasticity at stretching was noted. The publication noted an increase in the modulus value with an increase in the shell content in the composite, while in the studies presented in this paper, higher modulus values were observed for the composition filled with 5 wt.% unmodified peanut shells (for 5%U-PSF = 1467.4 MPa, 10%U-PSF = 977.4 MPa, REF = 1021.7 MPa). Comparable relationships were also obtained in the study of the effect of the addition of peanut shells mercerized with 10% NaOH solution to the LY55 Bisphenol A epoxy resin [55]. The highest tensile, flexural, and impact strength values were recorded for samples containing 50 wt.% shells with a particle size of 0.5 mm. At the same time, it was observed that with the increase in the size of the filler particles and their content in the composite, correspondingly lower values of strength parameters were recorded.

### 3.4. Results of SEM Analysis of Epoxy/Wood (Peanut Shell) Flour Composite

**Figure 6:**
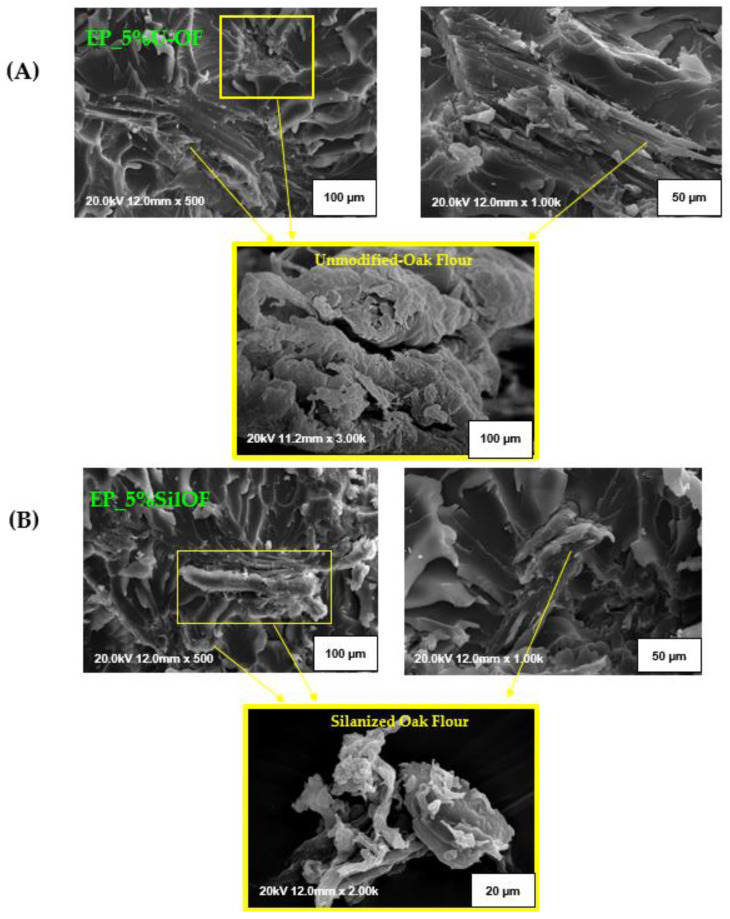
SEM micrographs of the impact fracture surface of the epoxy composites of EP_5%U-OF composition filled with 5 wt.% of unmodified wood flour (**A**), and EP_5%SilOF composition filled with 5 wt.% of silanized wood flour (**B**).

**Figure 7:**
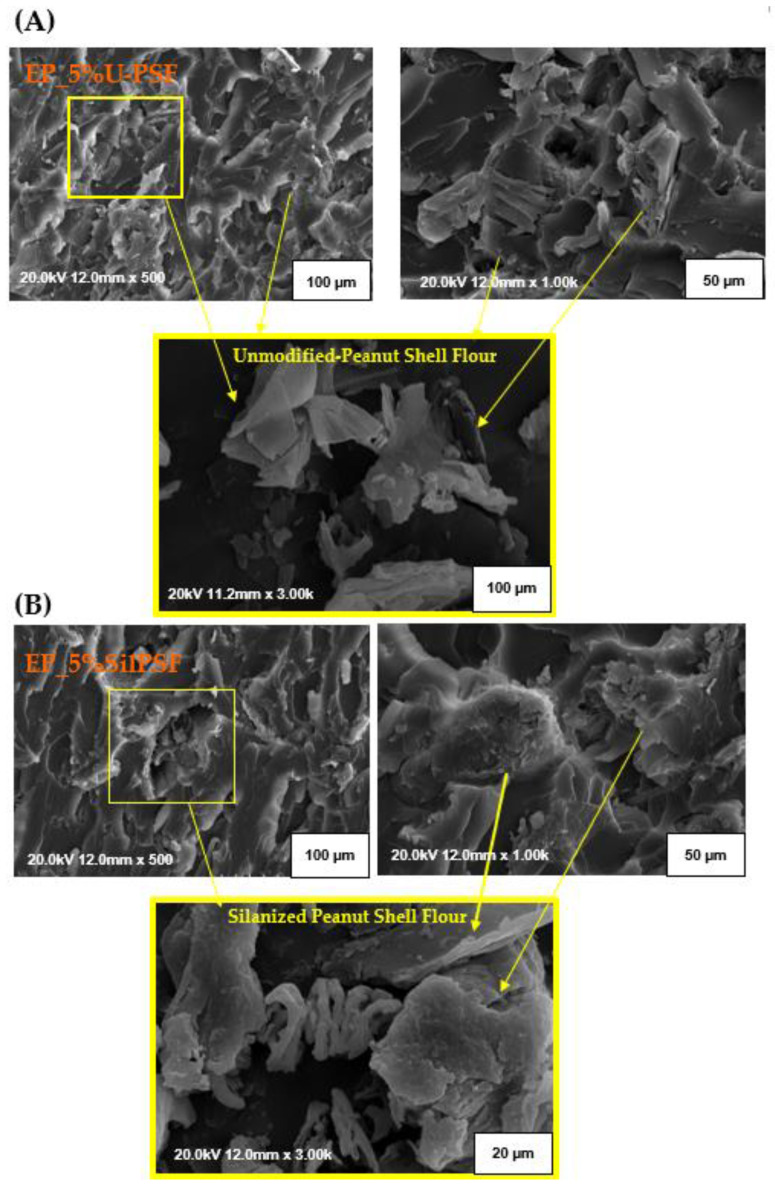
SEM micrographs of the impact fracture surface of the epoxy composites of EP_5%U-PSF composition filled with 5 wt.% of unmodified peanut shell flour (**A**), and EP_5%SilPSF composition filled with 5 wt.% of silanized peanut shell flour (**B**).

In the next stage composites containing oak and peanut shell flour were subjected to the morphology analysis, conducted by the SEM method (Figure 6 and Figure 7). Independently from the applied filler, within an entire volume of an analyzed sample containing unmodified lignocellulosic additive agglomerates of different sizes might be distinguished. They are unevenly distributed throughout the polymer matrix. Depicted agglomerates presumably influenced poor interfacial affinity between the components of studied composites leading to lower values of their mechanical properties, which was pointed out in Section 3.3. Additionally, the tendency to the creation of such agglomerates might result from the presence of the -OH groups, which were registered within the FT–IR analysis (Figure 4). -OH moieties have a higher chemical affinity for each other than for the polymer matrix. The surface of unmodified oak flour particles, which is presented in the SEM microphotographs (Figure 6) was originally rough. Additionally, the occurrence of numerous irregular strips might be observed, which probably was created during the earlier production stages. When comparing the observed oak surface with the unmodified surface of peanut shell flour particles (Figure 7), it can be noticed that captured surface is largely devoid of shreds that have been seen on wood waste. Additionally, as particles captured on microphotographs have been measured, it was noticed that for the unmodified isolated wood flour, most of the wood particles were about 23–39 µm. In the case of unmodified isolated peanut shell particles, a relatively large group of small elements of average dimensions of about 7–13 µm were found. These small units constituted the overwhelming majority and were separated by single larger particles with dimensions of approx. 19–42 µm. Differences in the size of grains introduced into the composite to a greater extent in combination with a much greater degree of coverage of unmodified particles by the residues of pectin, lignin, hemicellulose, and waxy substances, which corroborates the FT–IR results (Figure 4 and spectra interpretation included within Section 3.1), and may be an explanation of the previously found inferior strength properties of composites containing unmodified peanut shell flour.

The performed chemical modification by two-stage silanization process of lignocellulosic filler contributed to the partial removal of the residues of impurities, pectin, lignin, hemicellulose, and waxy substances, during mercerization—the first stage of performed modification. As well as, reducing, via combining the mercerization and silanization process, the hydrophilic nature of the additive by the elimination of hydroxyl groups contained in its structure, and on the other side introduction of the polar bridge-like silane structure which potentially contributed to an increase in the compatibility between epoxy matrix and lignocellulosic particles (Figure 3). The differences in the surface and dimensions of individuals between unmodified and modified particles (Figure 6 and Figure 7) are visible as a response of cellulose contained in the waste filler to the alkali treatment. As studied before [56], during the mercerization process a significant swelling of the additive (both oak and peanut shell particles) was observed. Such phenomenon was explained by the relaxation of the structure of cellulose consequently leading to an increase in the average content of the amorphous regions, which are more susceptible to further chemical modification, e.g., by the performed here silanization process. Silanized particles were characterized by a more complex structure, single units were more likely to merge into larger groups, probably due to the bridge-like structure related to hydrogen bonds between the silane groups, and the formation of siloxane bridges, as indicated within Section 3.1. The average particle size of silanized lignocellulosic particles was about 30–65 µm. Moreover, similarly to the modifications described in the literature [57] structural changes in the morphology of modified samples were observed. On the post-silanization particles, some irregular-shaped empty pores are visible. They probably showed up due to the partial removal of hemicelluloses and lignin. Nevertheless, the interface between the composite matrix and additive in the case of composites containing modified fillers (oak or peanut shell flour) is still noticeable. On the presented microphotographs, the pullouts of natural particles from the polymer matrix are visible. In the case of composites with modified additive, the filler is more coated with polymer matrix, making pullouts less observable than in the case of unmodified composites. Additionally, the observed results within the SEM microphotographs show a better coverage of the filler by the epoxy matrix resulting in better mechanical properties of composites with silanized additives.

## 4. Conclusions

The main purpose of the research described above was the comparison of the properties of epoxy composites filled with waste natural fillers in the form of oak or peanut flour. An additional goal of the performed studies was to develop another way of functional management for the reuse of natural waste, which globally is produced in relatively large quantities. To reduce the hydrophilic properties of lignocellulosic fillers two-stage silanization was performed. It was found that in the first step—the mercerization—a reduction of the content of hydroxyl groups associated with compounds, such as lignin and hemicellulose took place. Furthermore, the filler particles were purified from additional substances such as waxes, pectins, or other impurities deposited during the production process. On the other hand, the silanization process, both in the case of wood and peanut filler, resulted in an improvement of the compatibility between epoxy matrix and lignocellulosic particles via the introduction of bridge-like silane individuum, which has a positive effect on the interaction of the applied filler with polymer matrix, and potentially due to the chemical structure, additionally contributed to the curing process.

The addition of a filler to the epoxy matrix significantly affects the increase in the viscosity of the prepared composition. Different weight share of the filler in the compositions changes their mechanical properties. All compositions containing 10 wt.% of filler were characterized by generally poorer mechanical properties. The exception was 10%U-OF, which was characterized by the highest of all values of compressive strength (69.1 MPa) and Rockwell hardness (92.9 MPa). Compared with values registered for compositions filled with 5 wt.% of additive, a decrease in flexural strength by, respectively, 18.56% (composites with U-OF), 19.09% (composites with SilOF), 7.68% (composites with U-PSF), and 8.57% (composites with SilPSF) was observed. A similar effect was noted for tensile and compressive strength. The tensile strength for more content of silanized oak filler was lower by 6.4 MPa, while in the case of peanut shell flour, the deterioration effect was even bigger—the registered value was lower by 8.0 MPa. The compressive strength was reduced by 20.48% (composites with SilOF), 3.32% % (composites with U-PSF), and 2.50% (composites with SilPSF). Generally, it was observed that the performed modification resulted in better compatibility of lignocellulosic filler to the polymeric matrix. What is especially important to highlight here is that concerning pure BPA-based epoxy resin for the composites with oak filler, higher values of flexural and impact strength were recorded (respectively, flexural strength: 73.8 MPa—5%U-OF and 71.5 MPa—REF; impact strength: 15.82 kJ/m^2^—5%U-OF, 9.15 kJ/m^2^—REF). Registered differences in the effect noted for oak and peanut shell composites might potentially result from a higher content of impurities in the groundnut flour affecting the efficiency of the two-stage silanization and the bonding of the filler with the polymer matrix. Nevertheless, epoxy composites with such mechanical properties might be considered as broadly understood construction materials.

## Figures and Tables

**Table 1:** FT–IR analysis of waste bio-fillers.

Frequency [cm^−1^]						Associated Band	
Waste Oak Flour			Peanut Shell Flour				
U-OF	MOF	SilOF	U-PSF	MPSF	SilPSF		
3570–3100	3600–3035	3550–3060	3685–2993	3600–3018	3649–3007	-OH	*v* valencene
1724	-	-	1710–1528	1714–1532	1679–1581	-C-O	*v* valencene
-	-	1590	-	-	1581	-NH_2_	*δ* scissor
1596	1590		1501	1500	1498	-C-C-	*v* valencene
-	-	1230	-	-	1254	-C-N-	*v* valencene
1178–860	1172–930	-	1216	-	-	-C-O	*v* valencene
-	-	1030	-	-	1033	-Si-O	*δ* deformation
1170	1172	1028	1021	1019	1018	-C-O-C-	*v* valencene

Where: U-OF—unmodified oak flour; MOF—mercerized oak flour; SilOF—silanized oak flour; U-PSF—unmodified peanut shell flour; MPSF—mercerized peanut shell flour; SilPSF—silanized peanut shell flour.

**Table 2:** Composition of epoxy composites.

Epoxy Composition	Epoxy Resin	Hardener	Deaerator	Filler	
				Oak Flour	Peanut Shell Flour
REF	Epidian 6	Isophorone-diamine	BYK A530	–	–
5%U-OF				5 wt.% of unmodified oak flour	–
10%U-OF				10 wt.% of unmodified oak flour	–
5%SilOF				5 wt.% of unmodified oak flour	–
10%SilOF				10 wt.% of unmodified oak flour	–
5%U-PSF				–	5 wt.% of unmodified peanut shells flour
10%U-PSF				–	10 wt.% of peanut shells flour
5%SilPSF				–	5 wt.% of silanized peanut shells flour
10%SilPSF				–	10 wt.% of silanized peanut shells flour

**Table 3:** Mechanical properties of compositions based on Epidian 6 filled with wood or peanut shell flour.

Mechanical Properties	Tested Epoxy Compositions Based on Epidian 6 Filled with Wood or Peanut Shell Flour							
	Unmodified Oak Flour [wt.%]		Silanized Oak Flour [wt.%]		Unmodified Peanut Shell Flour [wt.%]		Silanized Peanut Shell Flour [wt.%]	
	5%U-OF	10%U-OF	5%SilOF	10%SilOF	5%U-PSF	10%U-PSF	5%SilPSF	10%SilPSF
Modulus of elasticity [MPa]	599.1 ± 8.4	1020.0 ± 436.4	671.2 ± 120.9	583.0 ± 167.4	1467.4 ± 825.8	977.4 ± 310.3	1618.2 ± 886.7	569.1 ± 240.1
Elasticity flexural modulus [MPa]	1795.7 ± 35.0	1736.8 ± 82.8	1832.5 ± 62.9	1552.3 ± 19.3	1446.0 ± 193.0	1572.3 ± 112.1	1352.3 ± 49.4	1159.0 ± 30.7
Rockwell Hardness [MPa]	77.2 ± 10.3	92.9 ± 1.2	74.1 ± 10.0	76.0 ± 2.0	68.1 ± 10.4	72.0 ± 12.7	86.6 ± 9.5	88.6 ± 14.3
Impact toughness [kJ/m^2^]	15.82 ± 1.79	7.09 ± 0.74	15.43 ± 2.70	8.36 ± 0.45	8.42 ± 0.72	8.00 ± 1.33	8.19 ± 0.76	9.81 ± 1.41

## Data Availability

Not applicable.

## Supplementary Materials

The following supporting information can be downloaded at: https://www.mdpi.com/article/10.3390/ma16083149/s1. Figure S1: The analysis of viscosity of bisphenol-A-based composition containing different amounts of natural filler in a form of waste oak flour or peanut shell flour. Figure S2: FT-IR spectrum of cured bisphenol-A-based compositions containing waste oak filler or peanut shell flour. Figure S3: Swelling of epoxy compositions based on bisphenol A. Figure S4: Microscope images of an uncured epoxy composition containing 5 and 10 wt.% of lignocellulosic filler in a form of unmodified/silanized oak or peanut shell flour. Figure S5: The analysis of the contact angle of the selected epoxy composition.
