# Supplementary material for: Modification of Epoxy Compositions by the Application of Various Fillers of Natural Origin
Source: Materials (Basel). 2023 Apr 17;16(8):3149. doi: 10.3390/ma16083149 (PMC10141959; doi:10.3390/ma16083149)
Supplement: Supplementary file 1 [file materials-16-03149-s001.zip › materials-2272443-supplementary-proofed.pdf]

Supplementary Materials

Modification of Epoxy Compositions by the Application of Various Fillers of Natural Origin

Anna Sienkiewicz * and Piotr Czub *

Department of Chemistry and Technology of Polymers, Cracow University of Technology, Warszawska Str. 24, 31-155 Cracow, Poland

* Correspondence: anna.sienkiewicz@pk.edu.pl (A.S.); piotr.czub@pk.edu.pl (P.C.)

Additional characteristics of prepared composites performed during the preparation of the composition for the curing process along with the post-curing characteristics are presented below. The supplementary files include the following figures along with the appropriate comment:

Figure S1. The analysis of viscosity of bisphenol-A-based composition containing different amounts of natural filler in a form of waste oak flour or peanut shell flour.

Figure S2. FT-IR spectrum of cured bisphenol-A-based compositions containing waste oak filler or peanut shell flour.

Figure S3. Swelling of epoxy compositions based on bisphenol A.

Figure S4. Microscope images of an uncured epoxy composition containing 5 and 10 wt.% of lignocellulosic filler in a form of unmodified/silanized oak or peanut shell flour.

Figure S5. The analysis of the contact angle of the selected epoxy composition.

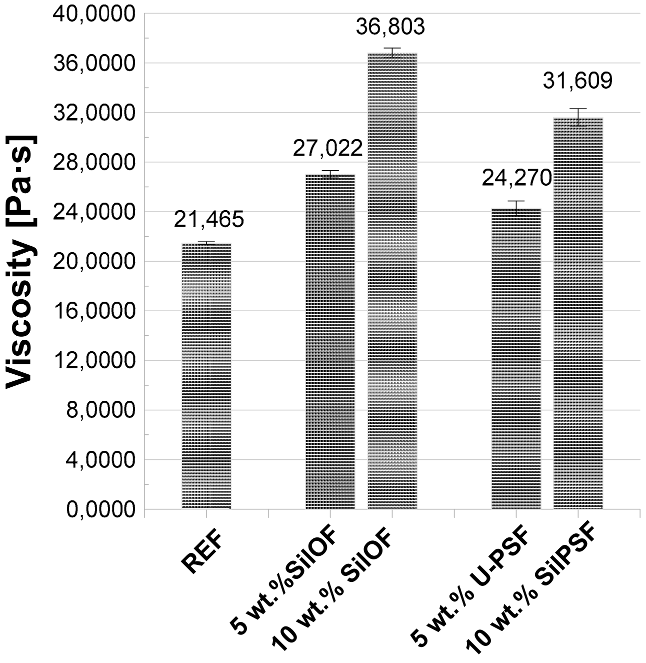

Figure S1. The analysis of viscosity of bisphenol-A-based composition containing different amounts of natural filler in a form of waste oak flour or peanut shell flour.

Assessment of the degree of cross-linking of the epoxy composition was carried out based on decay analysis bands of epoxy groups at 915 cm^{-1} wavelength, characteristic of group valence vibrations epoxy in the uncured resin. Below we present a summary of spectra recorded for individual compositions.

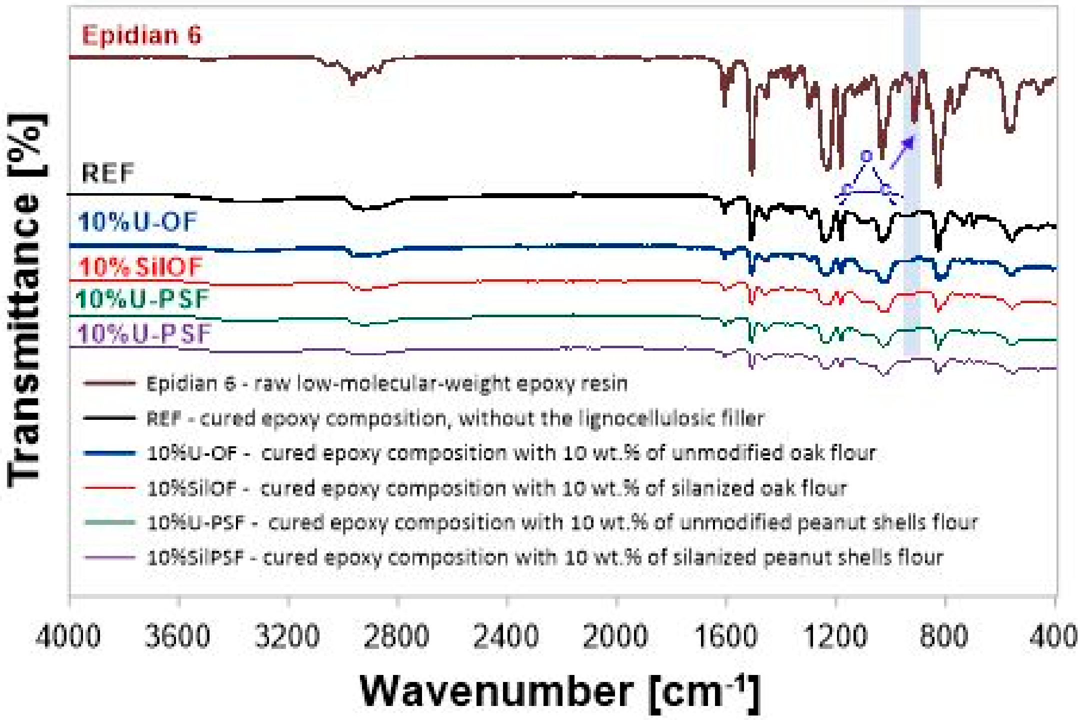

Figure S2. FT-IR spectrum of cured bisphenol-A-based compositions containing waste oak filler or peanut shell flour.

On the spectrum of bisphenol A low-molecular-weight epoxy resin a wide, characteristic band of stretching vibrations of the -O-H group in the wave number range of 3600-3000 cm^{-1} is visible. The next band, in the range of wavenumber of 3000-2700 cm^{-1} corresponds to the -C-H vibrations of the CH_2 and CH_3 stretching vibration of aromatic and aliphatic chains, respectively. On the registered spectrum there are also bands of vibrations characteristic for the -N-H groups, with wavenumbers respectively: v = 1606 cm^{-1} and v = 1581 cm^{-1}. These bending vibrations correspond to, used for curing, amine hardener. A signal at v = 1507 cm^{-1} comes most likely from -C-C stretching vibrations in the aromatic ring. On the other hand, the signal at v = 1368 cm^{-1} corresponds to stretching vibrations -C-N. While the vibrations -C-O-C correspond to signals at wavenumbers: v = 1235 cm^{-1} and v = 1021 cm^{-1}. Additionally, a signal indicating the -C-O stretching vibrations in the aromatic ring was recorded at v = 1180 cm^{-1}. Characteristic stretching vibrations of C–O–C and C–O attributed to ether linkage are confirmed by the presence of bands in the spectrum as a signal at v = 1232 cm^{-1} or 1082 cm^{-1}. Moreover, the signal of very low intensity originating from the oxirane ring is visible at v = 917 cm^{-1}. The band in the wavenumber range of 740-555 cm^{-1} most probably characterizes -C-H vibrations. Ramírez-Herrera et al. [*Ramírez-Herrera, C. A., Cruz-Cruz, I., Jiménez-Cedeño, I. H., Martínez-Romero, O.,*

Elías-Zúñiga, A., Influence of the epoxy resin process parameters on the mechanical properties of produced bidirectional [±45] carbon/epoxy woven composites, Polymers, 13(8), 1273, 2021] obtained similar results of the analysis of the chemical structure of epoxy resin.

Each of the spectra of the tested epoxy composites containing natural filler presents bands of the vibrations characteristic for amine -N-H (v = 1606 cm^{-1} and v = 1581 cm^{-1}). Signals, which indicate the vibrations of ether groups -C-O-C, are observed at wavenumbers v = 1235 cm^{-1} and v = 1021 cm^{-1}. On spectra registered for epoxy composites, no signals were observed at v = 917 cm^{-1}, which are characteristic of the vibrations of the oxirane group. This points to the properly carried out process of curing the epoxy resin, as a result of which the epoxide rings were opened by isophorone diamine. Additionally, when silanized additives were used bands characteristic of -OH groups (v = 3600-3000 cm^{-1}) were registered. These bands are characterized by lower intensity, compared to compositions containing unmodified additives. Vibration bands Si-O, recorded at v = 1150-1050 cm^{-1}, for silanized fillers most likely overlap with signals characteristic of -C-O-C vibrations. The presence of vibrations characteristic of individual fillers is not visible individually due to the overlapping of the signals coming from the matrix epoxy composites.

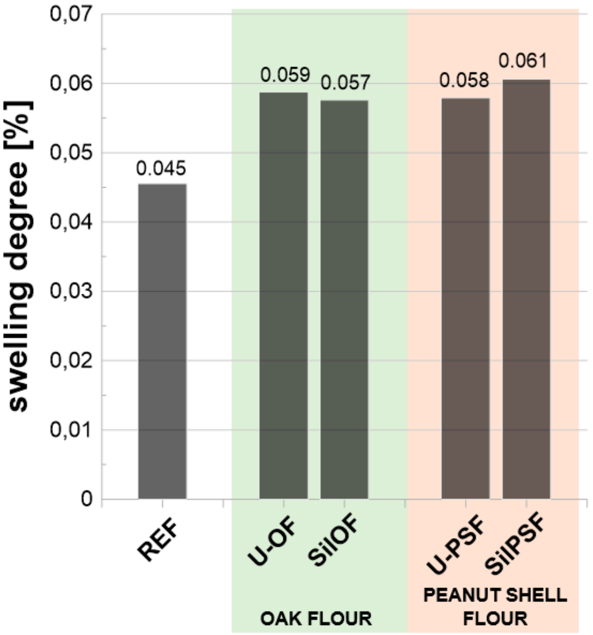

Figure S3. Swelling of epoxy compositions based on bisphenol A.

The results of the incubation of the epoxy compositions in water are shown below. The incubation has been performed on samples of obtained compositions with a thickness of approx. 1-2 mm and a weight of 40-90 mg. Samples were immersed in distilled water for 7 days. We have performed the analysis of swelling on the reference sample – the one without a filler, and compositions with the addition of 5 wt.% of filler of natural origin.

Water absorption is bound to the rate of its penetration into the polymer material. The polymer matrix shows sorption capacity, and thus absorbs water, which leads to swelling of the polymer and, as a result, to an increase in the weight of the samples. Cured epoxy resins are generally characterized by low water absorption, which usually does not change much. The greatest weight gain and thus the greatest differences in the weight of the samples after the first week of incubation occurred in the samples of composites containing the natural filler. The phenomenon of greater water absorption by composites containing lignocellulosic flour is related to its stronger absorption by natural fibers. Such a phenomenon is mainly related to the structure of cellulose. Cellulose molecules have free, polar hydroxyl groups that attract water molecules through hydrogen bonds, which in turn results in the accumulation of moisture in the cell wall and swelling of the fibers [*A, Ashori, S. Sheshmani (2010). Hybrid composites made from recycled materials: Moisture absorption and thickness swelling behavior. Bioresource Technology, 101(12), 4717-4720.*]. The introduction of modified lignocellulosic flour into the epoxy matrix resulted in a decrease in the amount of water absorbed in comparison with samples filled with unmodified natural filler. The obtained results additionally correspond with the images captured using an optical

microscope, which are presented below. As seen in these images, composites with modified lignocellulosic filler are characterized by good dispersion of the filler in the entire volume of the composite, individual wood particles are quite well separated from each other. The performed modification had an impact on better distribution of the filler within the polymer matrix and at the same time decreased the degree of agglomeration and the amount of water absorbed by the sample.

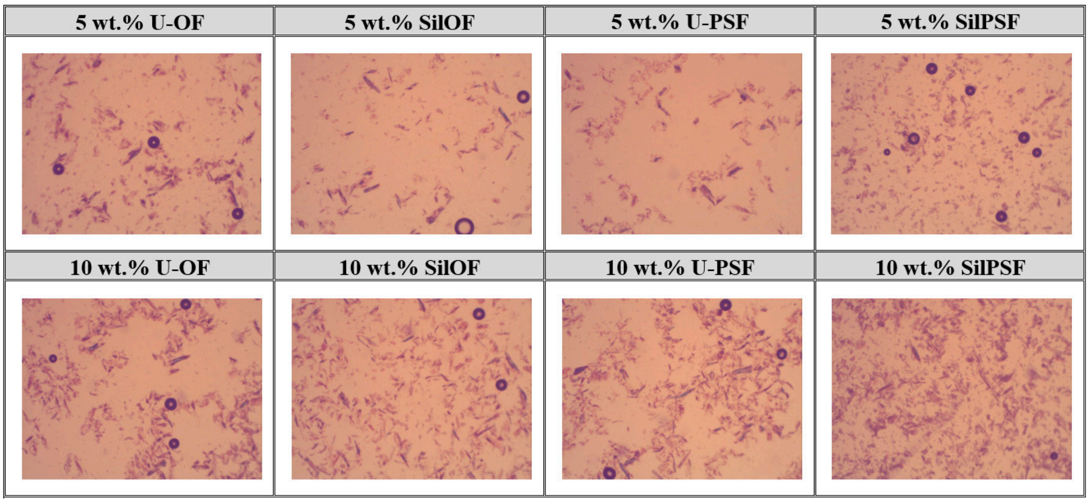

Figure S4. Microscope images of an uncured epoxy composition containing 5 and 10 wt.% of lignocellulosic filler in a form of unmodified/silanized oak or peanut shell flour.

At the same time, it was found that the introduction of peanut shell flour into the epoxy composition results in the highest values of absorbed water by the cured sample.

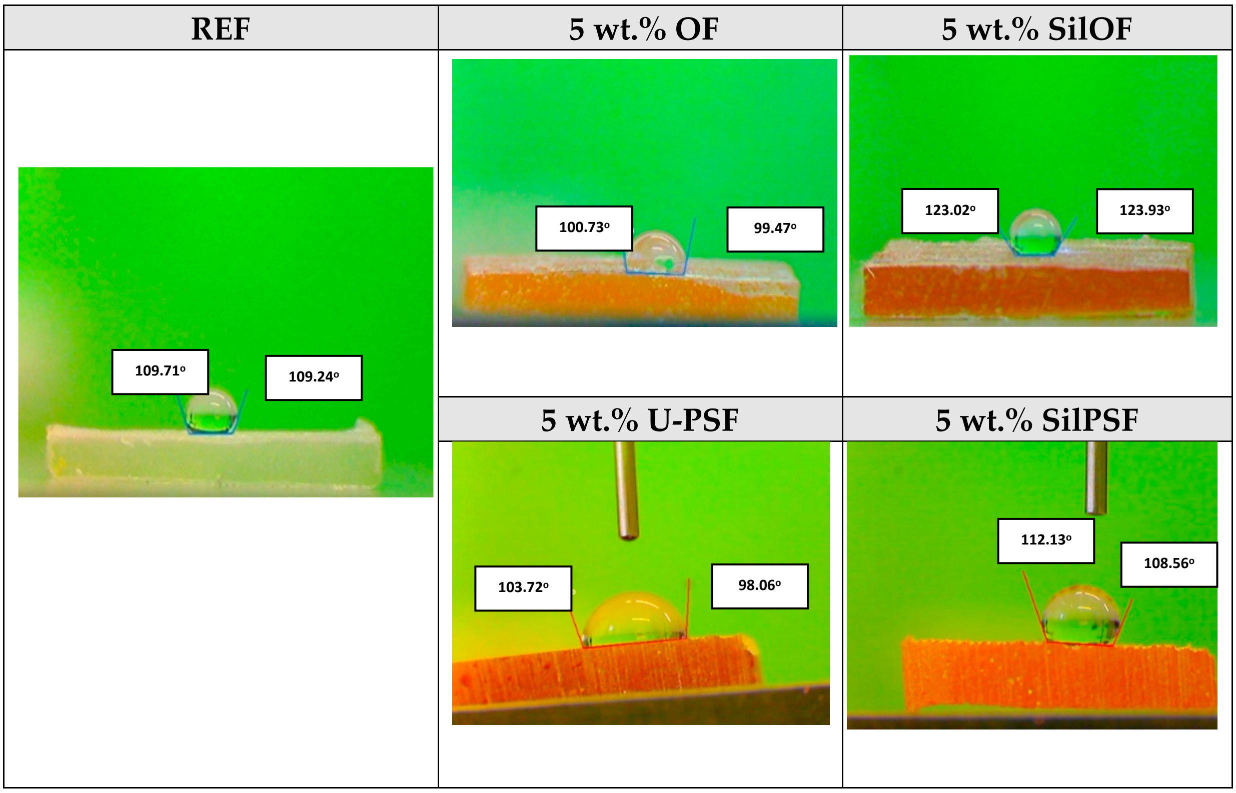

Figure S5. The analysis of the contact angle of the selected epoxy composition.
